# Cross-country comparison: does social democratic party power increase an employee’s perceived employability?

**DOI:** 10.3389/fsoc.2023.1212553

**Published:** 2023-10-03

**Authors:** Isabel M. Habicht

**Affiliations:** Department of Sociology, University of Wuppertal, Wuppertal, Germany

**Keywords:** employability, active labour market policies (ALMPs), social democratic parties, human capital investments, labour market insiders

## Abstract

Individuals strive to be highly employable, yet, we lack a uniform definition of ‘employability’. Within the labour market, employability can be seen as a product of individual human capital resources. However, this study argues that employability is also affected by the structure of the labour market and therefore also considers a country’s economic situation and political power to quantify employees’ perceived employability. Using data from the International Social Survey Programme, the Manifesto Project Dataset, and the International Labour Organization, this study uses a multilevel regression model with data from 30 countries. This paper disentangles the impact of individual careers and country policies (micro–macro linkage) on the perceived employability of their employees. At the individual level, initial education is the main predictor of employees’ current perceived employability, but vocational training is not. At the country level, the share of social democratic party power in each country, as a driver of active labour market policies, has a net effect on employee’s perceived employability, irrespective of their individual human capital investments. The generalisability of the findings is relevant to current debates about whether workers should become managers of their own careers or whether policymakers should take responsibility.

## 1. Introduction

Employability of individuals has become an increasingly important topic in recent decades for both employees and employers as well as society as a whole, responding to changing labour market structures. Although employability is a complex construct, in simple terms, it refers to the ability to realise career opportunities that encourage one to find employment (Fugate et al., 2004, p. 16). The term ‘employability’ has changed while adapting to changing career patterns from lifetime employment and loyal values to multiple employers with respect to flexibility and mobility − all under the premise of being employable and maintaining the workforce of a society. While research in Europe and the United Kingdom foregrounds employability in the context of social policy responsibility (e.g. McQuaid and Lindsay, 2005), US research focuses on employability by improving individual skills to respond to career changes (e.g. Fugate et al., 2004). The debate pivots on the question of whether workers need to become managers of their own careers or whether policymakers should assume the responsibility.

Drawing on this, employment policies target two groups with corresponding aims: first, individuals returning to work after unemployment (‘outsiders’)^1^; second, maintaining workers in employment (‘insiders’). While labour market promotion laws, such as active labour market policies (ALMPs), often focus on and ‘unambiguously benefit outsiders’ (Rueda, 2006, p. 388), this study particularly considers social democratic parties as drivers for ALMPs with respect to labour market insiders’ perceived employability, as they have received little attention yet.

While focusing on insiders, this study stresses the *duality of employability* and contains a structural and an individual dimension. The individual dimension emphasises employees’ human capital resources, while the structural dimension refers to national and regional economic conditions. Economic conditions vary across countries and periods, with corresponding effects on different ALMPs. Contributing to this, economists and policymakers are concerned about an efficient and stable economy within countries and are thus actively trying to maintain citizens in jobs to reduce unemployment. Because this objective involves at least two actors on two different soci(et)al levels – the micro and the macro level –, countries lack adequate ways to evaluate interdependencies. Hence, this study extends prior research that focuses solely on the individual level and underscores whether and why policy implementations need to be considered clarifying perceived employability.

## 2. Theoretical background and previous research

Research on and about the term ‘employability’ was already developed in the 1950s (e.g. Feintuch, 1955), but empirically studied only since the 1990s (Thijssen et al., 2008, p. 166).^2^ During this time, the definition of employability has changed considerably according to different time periods, but also structural social changes and work-related requirements. In this sense, linear and continuous career paths were replaced by ‘boundaryless careers’ (DeFillippi and Arthur, 1994; Arthur and Rousseau, 2001; for a critical review, see Sullivan and Baruch, 2009). This means that not only have careers been shaped differently, with multiple employers and higher levels of turnover and flexibility, but the demands placed on employees have also changed. Whereas in the past values such as loyalty were dominant, it is now common to have experience with more than one employer. The individualisation of careers thus also challenges how employees perceive their employability, as ‘employable’ workers are those with successful careers who can respond to changing labour markets (Carbery and Garavan, 2005).

Employability needs to be contextualised. Apart from a socio-economic perspective, however, some authors conceptually relate employability psychologically to individual capability and career development, while others also consider employers and organisational levels, or include social, cultural, and human capital investments to enhance employability (for a review, see Williams et al., 2016). In addition, research on employability has been undertaken by different disciplines such as psychology, educational studies, human resource development, management studies, and in different countries with different research foci. The juxtaposition of different perspectives makes it clear that increased employability is a goal in educational studies (through improved skills), management theories (through human resource management and strategies), psychological research (through career development), and a universal goal for a societal development to increase a country’s workforce and economic sustainability.

This paper focuses on and brings together two socio-economic perspectives that should not be considered separately: investments in human capital and labour market conditions that affect employee’s employability at two levels. This study therefore considers cross-country labour market structures and political power to increase the employability of employees, net of their own efforts to make such investments.

Before I turn to the conceptualisation of this study, however, I will explain why I focus on *perceived* employability of employees. Taking the perspective of employees and not policy makers, labour market insiders tend to perceive individual risks as an outcome of the political regime so that increased economic insecurity raises the likelihood of voting social-democratic parties (Walter, 2010; Rehm, 2011). This makes clear why social-democratic parties and their contribution on ALMPs are highly connected to how insiders perceive labour market security, risks, and their individual employability. For this reason, I take into perspective employee’s perception as it shapes their attitude on political regimes manifested by voting behaviour.

### 2.1. Perceived employability as an outcome of human capital endowments

At the individual level, an employee’s employability can be seen as a product of human capital endowment. Accumulating human capital through education and training leads to increased productivity (Becker, 1962, p. 11; Becker, 1993/1964, p. 17) but also to improved initial conditions on the labour market overall. Therefore, formal education and occupational skills are considered valuable investable ‘capital goods’ (Schultz, 1961, p. 2; Garavan et al., 2001). In other words, better educated and trained people have better chances in the labour market because high education signals effort and ability, thus increasing the perceived employability of employees, as can be seen by others (e.g. Berntson et al., 2006).

However, different types of human capital are relevant for employment outcomes. Whereas *general* human capital can also be applied to other employers, acquired *specific* human capital can only be used within the current employment, which lowers labour market returns. Investment in general and specific human capital therefore has different effects. While employees may benefit more from general human capital because it is transferable between employers, organisations may benefit more from investing in specific human capital because it contributes directly to organisational growth and success. While Becker (1993/1964) argued that investments in the employee’s specific human capital hold greater value for employers in highly competitive labour markets, other studies find that general human capital is more valuable as it translates to both the current and future employers (e.g. Rauch and Rijsdijk, 2013). Additionally, some authors conclude that specific and general human capital are ‘incentive complements’ (Kessler and Lülfesmann, 2006): Although it may be more beneficial for employers to invest in the specific human capital of their employees, it is not clear whether they can retain employees with higher investments if they cannot afford to pay higher wages accordingly. As a result, employees have an incentive to increase their general human capital in order to be attractive to other employers and organisations.

In contrast to this employability paradox from the employer’s perspective, which is reflected in mixed research findings, little is known about how employees’ general and specific human capital affect perceptions of their employability. Previous studies have not been able to distinguish between specific and general human capital as having different effects on employability; they measure job experience (‘job tenure’, e.g. in Berntson et al., 2006) but cannot distinguish whether previous job experience is transferable to the current employer as well. From the perspective of a labour market insider, the question is therefore: Is general human capital, through transferable job experience, more beneficial to an employee’s perceived employability than specific human capital, which is employer-specific and rarely transferable?

In addition, the lifelong learning approach encourages workers to continue training beyond their initial education in order to improve their skills and further invest in their human capital, which may also increase their employability. In this sense, this paper focuses on three different human capital resources that are strongly related to employment trajectories: human capital through initial schooling, through job experience that may or may not be transferable to other employers, and through further vocational training activities that aim to increase labour market utility in the long run. All dimensions are related to employees’ perceived employability, but it is not clear – from the theoretical underpinning and empirical evidence – how they directly influence perceived employability, although all resources are aimed at increasing labour market utility. In other words, it is unclear how different types of human capital investments improve an employee’s employability, which this study aims to address at the micro level.

### 2.2. Perceived employability as an outcome of the labour market structure

Although studies agree that education and skills boost employability (Berntson et al., 2006), others strongly argue that microeconomic variables are ineffective in measuring employability unless macro-level variables are simultaneously considered (*cf.* Caroleo and Pastore, 2001). This is because expenditure on ALMPs, such as training, employment incentives, or job creation, varies between countries. Accordingly, priorities and support are distributed differently across countries and political regimes, which in turn affects employees and their training opportunities and perceived employability. From a macro perspective, countries with a higher share of social democratic parties also have well-developed ALMPs (Huo et al., 2008), as ‘employment-friendly’ policies originate from social democratic governments.

But why is this so? The reason traces back to policy preferences that differ before and after the ‘activation turn’, which shifted towards neoliberalism at the end of the 1990s and beginning of the 2000s (e.g. Green-Pedersen et al., 2001; Nelson, 2013). The transformation of social democracy, also known as the ‘third way’, includes decommodification that frames economic competitiveness turning into individual responsibility for labour market participation. ‘Flexicurity’ became one of the leading keywords, regarded as the new workforce ideal (*cf.* Nelson, 2013, p. 261). Social democratic parties therefore favour labour market insiders and their participation rather than outsiders (as can be seen by Rueda, 2005, 2006). In this sense, ALMPs are somehow a bi-product of social democratic parties to strengthen the emphasis on individual responsibility.

However, there are different conceptualisations of when a country can be classified as social democratic. One of the most prominent is Esping-Andersen’s (1990) typology of welfare states, which distinguishes between conservative, social democratic and liberal welfare state regimes in different countries. With reference to the degree of decommodification, social stratification, and private and public aspects (family and role of the state) of a country, the classification is used to characterise a country’s welfare regime in an ideal-typical way. However, rather than focusing on different welfare regimes, this study focuses on differences in the political composition of countries and their economic situation, as both are thought to act as drivers of ALMPs. This methodology enables a more precise analysis of differences among countries that goes beyond Esping-Andersen’s typology, which is limited to specific countries.

Nevertheless, empirically, the relationship between social democratic parties and ALMPs is not that clear. While Huo et al. (2008) find social democratic parties as the main predictor of ALMPs, Rueda (2006) demonstrates non-significant or even negative effects of social democratic governments. Bonoli (2010) argues that although country- and time-specific political regulations hardly affect ALMPs, most of the social-democratic parties’ preferences conform with human capital investments and occupation. But what does the implementation of activating labour market policies actually look like on the employee and employer side?

Although the elaboration of ALMPs varies across countries and governments, ALMP’s implementations can be summarised by (1) employer incentives like wage subsidies, (2) job search assistance, (3) programmes to overcome short-time unemployment and keep people busy and (4) human capital investments and training (*cf.* Bonoli, 2010; Card et al., 2010; Filges et al., 2015). However, while categories (1) to (3) target labour market outsiders, human capital investments as the ‘classic’ component of ALMPs are particularly beneficial for labour market insiders. Therefore, the European Commission has begun to implement a more flexible and longer-term strategy based on lifelong learning to boost employability through human capital investments (Council of the European Union, 2015). This development shows the importance of policy implementation to improve the employability of employees, which is why measures at the macro level should be considered in addition to those at the micro level.

Empirical evidence supports the micro–macro link assumption. Beyond the borders of the EU, McQuaid and Lindsay (2005) give examples advocating employability-focused ALMPs of the United Nations (UN) and the OECD. Notably, job training makes up the biggest category of spending on ALMP programmes (*cf.* Bernhard and Kruppe, 2012), together with work experience (Kluve, 2006). Kluve (2006) emphasises the broad heterogeneity in ALMPs across countries, mirrored in different funding levels of vocational training programmes. Contrary to Austria, the United Kingdom, and Scandinavian countries, Germany and Spain legislatively oblige employers to fund vocational training programmes to improve employees’ skills (Bollérot, 2001). The Nordic states merely follow a model of ‘activating people’, while Britain, by contrast, implements a ‘welfare to work’ model. The policy implementations demonstrate the direct link of structural changes on workers aiming to improve employees’ work skills to make them more competitive and employable.

Therefore, I expect social democratic parties to promote ALMPs within countries that increase human capital through further training programmes. While many studies focus only on the macro-level to evaluate ALMP programmes across countries (Kluve et al., 2007; Huo et al., 2008; Bonoli, 2010), I aim to measure how the share of social democratic parties that promote ALMPs affects an individual’s perceived employability alongside their improved education or training. Few studies focus on a micro–macro linkage to consider labour market conditions along with individual variables, targeting only specific countries (e.g. Berntson et al., 2006; De Cuyper et al., 2011). While this focus provides an interesting insight into the labour market and workforce in specific countries, it misses the opportunity to examine how employees and policies interact across borders.

## 3. Data and methods

For the analyses, I use data from questionnaires based on the International Social Survey Programme (ISSP), containing the rotating module ‘Work Orientations IV’ which were last collected between 2015 and 2017 (Jutz et al., 2017). I exclude countries with missing information on the employee level so that the final data set includes 30 countries (for an overview, see Supplementary Table A). The final sample consists of 16,438 employees: 8,013 males and 8,425 females (15–65 years) currently in paid work.^3^

### 3.1. Variables

The *outcome variable* of my analysis measures employees’ perceived employability (insider perspective). The ISSP data provides three indicators to operationalise employability: respondents’ value on the labour market, expected exit, and job insecurity (Jutz et al., 2017, see Supplementary Table B for further details on the indicators). After testing the reliability between the three items used, a low Cronbach’s alpha score (α = 0.20) suggests a lack of internal validity. Because of this ‘under-correlation’ and due to the nature of latent variables, I conducted an Exploratory Factor Analysis (EFA) based on a common factor model (Brown, 2015). The factor analysis aims at determining the number of factors according to the observed variables (see Supplementary Table B for factor loadings). The result is a two-factor model that rejects the underlying one-factor assumption of employability supported by the ISSP. Because the factor loading is lower than 0.30, I exclude the variable that indicates ‘job insecurity’ for further analysis. In conclusion, I pursue a one-factor solution based on two items covering ‘employability’ (see Supplementary Table B). The current analysis reveals an acceptable Kaiser-Meyer-Olkin value (KMO) of 0.50 (Kaiser and Rice, 1974). For subsequent analyses, I use predicted factor scores as the outcome variable.

#### 3.1.1. Micro-level variables

On an individual level, I first add the *education level* (based on ISCED-97) as human capital. For international comparison, I recode the educational levels by (1) no formal education and primary school (elementary education), (2) lower secondary, (3) upper secondary (to provide skills relevant to employment), (4) post-secondary, but non-tertiary (programmes to provide learning experiences that build on secondary education), (5) lower level tertiary (Bachelor and Master or equivalent), and (6) upper level tertiary (doctorate/PhD, post-doctorate qualification).

Next, I measure specific human capital through people who could use almost none (1) of their prior *job experience* for the current job (rarely transferable) and general human capital through people who can use a little (2), a lot (3) to almost all (4) of their prior job experience for the current job (highly transferable).

To focus on a life-long learning approach as human capital that increases employees’ employability, I include a variable that covers respondents participating in *further vocational training* over the past 12 months (with answer options yes or no).

As said in the introduction, employability is a complex construct covering different notions depending on different levels: individual, organisational or industrial. With respect to this, I add several control variables to include information on individuals’ social and demographic characteristics, work attitudes, and employment trajectories: age, gender, relationship status, place of living, occupation, employment status, and respondents’ meaning of work. I group respondent’s *ages* in 10-year intervals, ranging from age 15 to age 65. I further use dummy variables to control *gender* (female = 1), and *employment status* (full-time vs. part-time contracts). For *relationship status*, I use categories for being single, in a relationship, or separated. I add the *place where respondents live*, categorised in urban area, small town, and rural area. *Occupational classes* are based on the International Standard Classification of Occupations (ISCO-08).^4^ I add these variables to control for job availability and access to the labour market. I also include a variable representing individuals’ work orientation, their *meaning of work* (‘a job is a way of earning money—no more’). Based on the possible responses, I used three categories separately for ‘(strongly) disagree’, ‘neither agree nor disagree’, and ‘(strongly) agree’.

#### 3.1.2. Macro-level variables

On the country level, I use three indicators that affect an employee’s employability. First, I add the share of *social democratic parties* in each country (see Supplementary Table A ‘Last vote’ and Supplementary Table C3). Contrary to a pre-classification, e.g. by the typology of Esping-Andersen, I summed the share within a government classified as ‘socialist parties’ or ‘social democratic parties,’^5^ provided by the Manifesto Project Dataset (Volkens et al., 2017). I also add the *labour force participation rate* and *labour productivity rate*.^6^ Provided by the International Labour Organization (ILO), I imputed the data at the time the survey started within each country (see Supplementary Table A, ‘Date of imputation’). First, the labour force participation rate expresses the percentage of working-age persons in the labour force (economically active population)^7^ in the total working-age population. Therefore, the labour force participation rate indicates the composition and size of human resources within each country (*cf.* International Labour Organization I, 2015). Second, labour productivity is linked to GDP in each country.^8^ Thus, labour productivity relates to ‘the efficiency and quality of human capital in the production process for a given economic and social context’ (International Labour Organization II, 2016, p. 1). All three macro-variables were standardised.^9^

### 3.2. Analytical procedure

For the analysis, I use two-level hierarchical linear modelling (Snijders and Bosker, 1999; Hox et al., 2010), also referred to as multilevel regression. The model implies a two-level regression model with employees as the first-level unit and countries as the second-level unit. Multilevel design structures are hierarchical and can therefore deal with employees being clustered in countries. Thus, the model combines within-group and between-group variation that allows to estimate both between- and within-cluster variability of an outcome variable, taking into account intraclass correlation (ICC) and cluster size (Hox et al., 2010).

First, I estimate a *variance-component model* (empty, unconditional model) without any covariates. By assumption, the variance-component model explains no variance in the outcome variable, while the intraclass correlation calculates the variance explained by the country structure (analogous to the coefficient of determination *r*^2^). Both the ICC and the cluster size meet the required criteria in the current analysis given an ICC^9^ of 0.048 and a total number of countries of M = 30 (*cf.* Kreft et al., 1998; Maas and Hox, 2005). Second, I estimate several *random intercept models* in a stepwise procedure.^10^

Because of the (small) size of 30 countries on level 2, I use restricted maximum likelihood (REML). Whereas REML yields a more realistic model (Hox et al., 2010, p. 41), maximum likelihood (ML) underestimates variance components (Snijders and Bosker, 1999, p. 56; Raudenbush and Bryk, 2002, p. 53).

## 4. Results

### 4.1. Descriptive results

**Figure 1:**
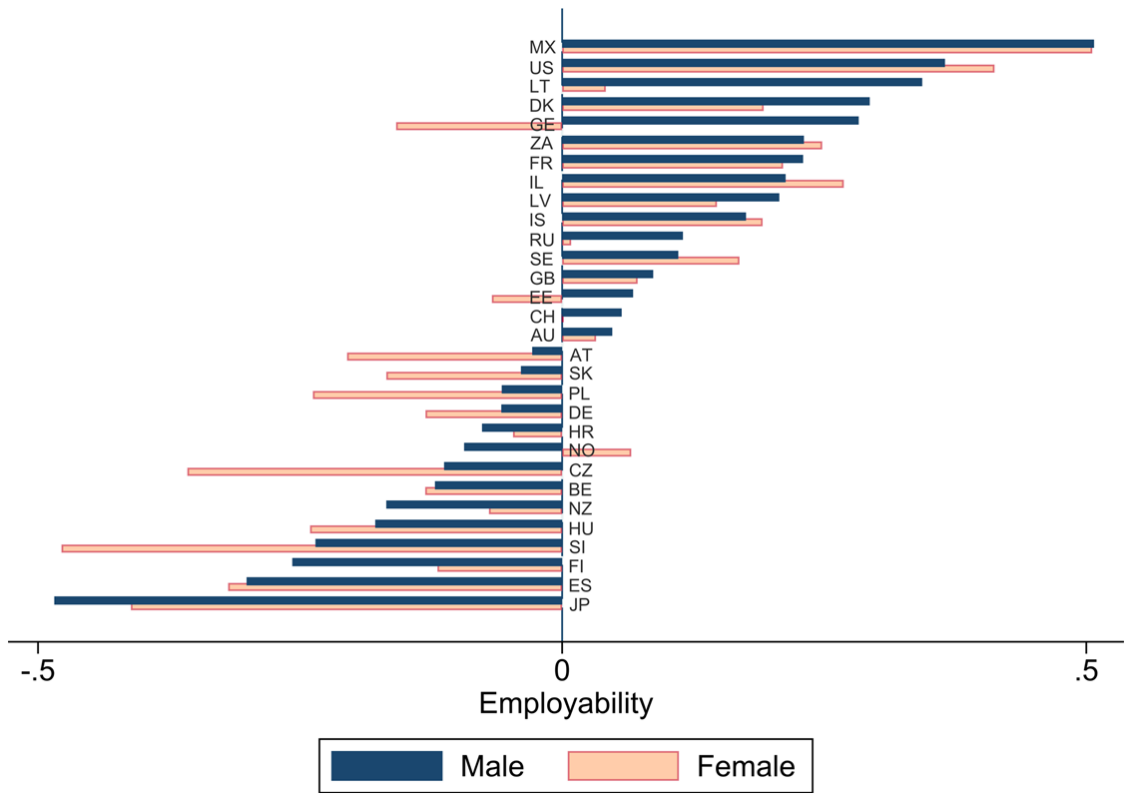
Mean perceived employability by country and gender.

Figure 1 shows the mean perceived employability (predicted factor scores) for each country, separated by gender. The employability scores range between ±0.5 points across countries (centred at the mean; min = 1.5 and max = 2.7; standard deviation = 1). Countries with positive employability scores are, for example, Mexico (MX) and the United States (US). Notably, in countries with positive scores, employees’ perceived employability varies less across gender. Exceptions to this are Georgia (GE) and Estonia (EE) – male employees perceive employability higher than female employees. On the contrary, the values for women and men diverge more in countries with below-average employability (< 0). In Norway (NO), however, women report over-average scores while men have under-average employability scores.

### 4.2. Testing the multilevel model of employability

The multilevel model in Table 1 includes five models in a stepwise procedure (control variables not shown here, see Supplementary Table H for full models). Model 1 specifies an empty model, whereas Model 2 and Model 3 add variables on the micro-level, and Model 4 and Model 5 add variables on the macro-level.

**Table 1:** Multilevel results of employee and country-level variables on perceived employability (control variables not shown here).

	(1)	(2)	(3)	(4)	(5)
	Variance – component model	Random intercept models			
Constant	0.01	0.71^***^	0.72^***^	0.72^***^	0.72^***^
	(0.04)	(0.05)	(0.06)	(0.06)	(0.06)
Fixed: Level 1					
Education					
Upper level tertiary		Reference			
Lower level tertiary			−0.06^*^	−0.06^*^	−0.06^*^
			(0.02)	(0.02)	(0.02)
Post secondary, non-tertiary			−0.12^***^	−0.12^***^	−0.12^***^
			(0.03)	(0.03)	(0.03)
Upper secondary			−0.15^***^	−0.15^***^	−0.15^***^
			(0.03)	(0.03)	(0.03)
Lower secondary			−0.17^***^	−0.18^***^	−0.18^***^
			(0.03)	(0.03)	(0.03)
No formal education^a^			−0.25^***^	−0.26^***^	−0.26^***^
			(0.05)	(0.05)	(0.05)
Job experience					
Almost none		Reference			
A little			0.15^***^	0.15^***^	0.15^***^
			(0.03)	(0.03)	(0.03)
A lot			0.13^***^	0.13^***^	0.13^***^
			(0.03)	(0.03)	(0.03)
Almost all			0.14^***^	0.14^***^	0.14^***^
			(0.03)	(0.03)	(0.03)
Further vocational training			−0.01	−0.01	−0.01
			(0.02)	(0.02)	(0.02)
Fixed: Level 2					
Left party power (std)				0.07^*^	0.10^**^
				(0.03)	(0.03)
Participation (std)					0.11^**^
					(0.03)
Labour productivity (std)					−0.01
					(0.04)
Random part					
Variance components					
Individual	0.93	0.85	0.85	0.85	0.85
	(0.01)	(0.01)	(0.01)	(0.01)	(0.01)
Country	0.05	0.04	0.04	0.03	0.03
	(0.01)	(0.01)	(0.01)	(0.01)	(0.01)
AIC	45589.34	44225.65	44214.30	44217.35	44221.45
BIC	45612.47	44410.63	44468.64	44479.40	44498.91
M (country)		30	30	30	30
*R*^2^ (individual)^b^		0.092	0.095	0.099	0.108
*R*^2^ (country)^b^		0.173	0.172	0.252	0.430
*df*	0	21	30	31	33
N (individual)	16,438	16,438	16,438	16,438	16,438

Note: Estimations from the random intercept model with individual- and group-level effects (REML). Standard errors in parentheses. Control variables: age, gender, relationship status, place of living, occupation, employment status, and respondents’ meaning of work. ^*^p < 0.05, ^**^ *p* < 0.01, ^***^ *p* < 0.001. ^a^Includes primary school. ^b^Variance explained by proportional reduction in prediction error (Snijders and Bosker, 1999, pp. 99–105; Snijders and Bosker, 1994, pp. 350–354).

After estimating an empty model (Model 1), Model 2 includes control variables on the micro-level. Because a (standardised) factor variable measures employability, I interpret results according to sign and magnitude rather than the actual effect size.^11^

Model 3 adds explanatory variables (investments in human capital) at the individual level. First, lower educational categories correlate with negative employability scores linearly as compared to the highest educational level (university degrees). That is, individuals with no formal education perceive the lowest employability (about 0.3 standard deviations below the mean) compared to highly educated workers. This statistically significant result shows the net effect of higher initial education, which is not surprising *per se*, but also cannot be attributed to, for example, a higher perceived employability of younger people or to a particular gender (as suggested for some countries in Figure 1), as both are controlled for. Second, employees with little to a lot of transferable previous job experience have higher employability scores than those with no (or almost no) previous job experience. That is, the transferability of prior work experience is essential to promote current employability. Third, participation in further vocational training does not significantly increase the perceived employability of employees.

Models 4 and 5 add country-level variables (full model). Model 4 shows that the share of social-democratic party power increases an individual’s perceived employability net of other variables (*b* = 0.07, *p* < 0.05), Model 5 adds macroeconomic covariates, i.e. labour market participation and labour productivity rates, which cover part of the influence that is genuinely attributable to social democratic parties (*b* = 0.10, *p* < 0.01). Without controlling for macroeconomic variables, the impact of social democratic party power on perceived employability is underestimated. Taking country-specific labour market factors into account shows an increased net effect of social democratic party power on employability across countries.

By looking at the variance explained across the models (*R*^2^), I use a proportional reduction in prediction error for level 1 and level 2 variables (Snijders and Bosker, 1999, pp. 101–105). Model 3 includes individual-level variables solely so that 9.5% of the explained variance is located at the individual level, while 17.2% is located at the country level. These results suggest that individual-level variables explain 17.2% of the country level’s variance and thus indicate a composition effect (between-group variance). That is, countries differ in their employees’ composition concerning individual-level variables. By adding country-level variables, the explained variance at the country level increases from approximately 17% (Model 3) to 43% (Model 5). The variation’s amount provides evidence that macro-level variables contribute to predict an employees’ employability.

### 4.3. Robustness checks

For robustness checks, I first use a multilevel model estimated on REML, including standardised level 1 and level 2 variables. Standardisation preserves the variance proportions in random intercept models but simplifies the interpretation of the coefficients (Hox et al., 2010, pp. 59–63). At the micro level, the standardised coefficient of education is equal to 0.060 (*p* < 0.001), that of job experience is equal to 0.027 (*p* < 0.001), and that of further vocational training is equal to −0.009 (*p* > 0.5). Relatively, education still exhibits the highest impact on employability.

Second, due to factor scores as an outcome variable, I ran two HLMs for both (ISSP) indicators of employability separately: expected exit and respondents’ value in the labour market. The best model fit is achieved by using the factor variable as the outcome (compared by AIC and BIC).

Third, I consider sample weights for a full multilevel regression analysis of micro- and macro-level variables. The ISSP’s weights are calculated on level 1 variables and therefore may underestimate cross-country comparison. Overall, unweighted results are robust and the model fits best for factor analysis for full multilevel modelling, including all explanatory as well as control variables at employee and country level.

Fourth, I add a score based on indicators of the Employment Protection Legislation (EPL) for each country, provided annually by the ILO (*cf.* International Labour Organization, 2015). Notably, EPL has a statistically significant but somewhat negative impact on the employability of women (see Supplementary Table F). Because the ILO does not provide EPL information for all countries within the dataset, I had to reduce the sample size. Because results hardly changed, I neglected to include the EPL index in the main analyses.

Finally, I run the full model separately for female and male employees (Supplementary Table H). School education seems to affect women’s perceived employability more strongly than men’s. In addition, prior work experience also seems to impact women’s employability more substantially. While even a little transferable work experience (as opposed to almost none) can increase women’s perceived employability twice as much as men’s (0.2 vs. 0.09), vocational training programmes do not increase employability scores for either women or men. In turn, social democratic parties within a country help women and men fairly equally in terms of employability. Additionally, the study shows a valuable impact of political and economic structure on female employees’ perceived employability, because 49 percent of the variance can be explained by considering social-democratic parties across countries and labour markets, compared to only 37 percent for men.

## 5. Discussion

In this study, I examined how investments in human capital and social democratic parties as drivers of ALMPs affect employees’ perceived employability across 30 countries. While I find higher education as significantly beneficial for employees’ perceived employability across countries, the share of social democratic parties in the countries also independently contributes at the macro level.

On an individual level, first, higher *initial education* as a human capital investment increases employability, which is coherent with other studies (e.g. Berntson et al., 2006). Whereas this finding hardly surprises, this study additionally shows that initial education, compared with work experience or vocational training, impacts future employability most decisively. In other words, along with initial education, university degrees reveal relatively long-term effects throughout people’s working lives, emphasising the importance of initial education to determine future employment activities and career paths.

In terms of previous *job experience*, the second dimension of human capital, the results of this study show that the perceived employability increases as soon as previous job experience is transferable to the current employer. This finding clearly indicates that general human capital plays a more crucial role in perceived employability – although it does not make a difference whether little or almost all of the previous experience is transferable to the current job. This result is confirmed by other studies, e.g. Rauch and Rijsdijk (2013), and contributes to the debate on employability with new insights: As flexibility and mobility are part of the characteristics of contemporary labour markets, it seems reasonable that general human capital, provided by the transferability of job experience, is more important for employees than specific human capital, which binds them to one employer.

The third dimension of human capital at the micro level relates to the lifelong learning approach, which encourages individuals to increase their investment in human capital through *further vocational training* in order to improve their employability; an approach that has recently gained attention and is gaining ground in the economy and among employers. While recent studies have found a positive association between lifelong learning, mediating human capital and employability (Nimmi et al., 2021), I do not find significant effects of further vocational training on the employability of employees. Thus, initial higher education has a more favourable impact on future employability than continuing vocational training linked to a lifelong learning approach, a finding similar to that of Korpi et al. (2003). This finding is probably the most surprising one in the context of ALMPs, as one of their pillars is to increase employability through further training programmes (*cf.* Bonoli, 2010; Card et al., 2010; Filges et al., 2015). According to Ceschi et al. (2021), the implementation of lifelong learning measures by policymakers can vary between countries, consequently making it challenging to compare employability improvement across countries. However, the conclusion of this study are in line with studies focusing on the benefits of vocational training for labour market *outsiders* transitioning into employment: research across different countries has shown that the positive long-term effect of vocational training programmes is absent (Nivorozhkin, 2005; Hujer et al., 2006; Hirshleifer et al., 2016). In general, these findings highlight the necessity of reforming lifelong learning approaches by implementing vocational training measures that aim to support long-term employment for both labour market insiders and outsiders. According to a study on youth unemployment in developed countries conducted by Zimmermann et al. (2013), there is a need to synthesise education and training programmes more effectively. Consistent with this, employability is already referenced in the Bologna Process to address the ‘changing dynamic between higher education and the labour market that had already occurred over the past decades’ (Sin and Neave, 2016, p. 1448). As a limitation of this study, however, the current study’s findings are restricted as it only observed employee’s participation in vocational training within the last 12 months.

On the country level, the share of *social democratic parties* within the government correlates positively with perceived employability across countries, even when controlling for employees’ human capital investments. Social democratic parties aim to improve labour market integration and continued employment through the implementation of more developed ALMP programmes (Huo et al., 2008). Although some authors do not find evidence for this relationship (*cf.* Rueda, 2006), this study indicates the importance of a country’s political composition in relation to their employee’s employability.

Unlike other authors who focus on different employment situations (e.g. Cortès-Franch et al., 2019), I decided against operationalising social democracy through Esping-Andersen’s welfare state typology. From this perspective, it is reasonable that my results are not consistent with Esping-Andersen’s ideal-typical conceptualisation, especially because the typology is limited to certain countries, while this study goes beyond this selection. However, by focusing on labour market structures rather than welfare states, I agree with Brown et al. (2003, p. 122) who conclude that employability may indeed mean being employable, but still ‘not be[ing] in employment’. Although the unemployment rate is correlated with the power of social democratic parties across countries, some countries have a higher proportion of social democratic parties but still face higher unemployment rates. This is more often the case in emerging economies such as South Africa. For this reason, the share of social democratic or socialist parties is only a partial expression of ‘social democracy’.

Finally, one last remark about both labour market insiders and outsiders. For the purpose of this study, the term ‘outsiders’ refers to those who are unemployed. However, it should be noted that employees with insecure and low-paid jobs could also be classified as outsiders (rather than insiders, as I have done in this study). This includes those who ‘hold jobs characterized by low salaries and low levels of protection, employment rights, benefits, and social security privileges’ (Rueda, 2005, p. 62). Nevertheless, I have opted for a broader definition of ‘insiders’ because of the theoretical ambiguities and empirical difficulties in distinguishing different gradations in this group. This study has revealed a positive association between social democratic parties acting as drivers for ALMPs and the employability of labour market insiders, including workers in precarious positions. Future research should concentrate on job compositions and levels of insecurity concerning ALMPs. This study does not ascertain if labour market outsiders (the unemployed) benefit from political and economic activity in the same way as labour market insiders. Nevertheless, the foremost message of this article should be that the share of social democratic parties in the government is positively associated with improving the employability levels of all labour market insiders (employees), irrespective of their educational background, transferability of skills and vocational training, adhering to their initial objectives.

What are the political implications of this study for the question of whether workers should become managers of their own careers or whether policymakers should take responsibility? This study shows that it is not solely on individuals but also on policymakers to respond to changing labour market conditions that are relevant for individual’s employability. Governments should provide *conditions* that increase human capital through better schooling or lifelong training opportunities, along with a stable economy and developing markets. Aware of this responsibility, employability policies were implemented in the 1990s in the member countries of the Organisation For Economic Cooperation and Development (OECD) in Europe and other regions (for an overview, see McQuaid and Lindsay, 2005). In this regard, the authors refer to Gazier (2001, p. 202), who points out that employability now involves interactions between individuals, other actors, and labour market conditions. Notwithstanding, individual-centred perspectives acknowledge employees as ‘active agents who initiate improvement in their work situations’ (Fugate et al., 2004, p. 15). Due to the multifaceted nature of employability, both (and other) perspectives constitute the construct of employability.

This study aims to bring together different socio-economic perspectives into one integrated concept of employability, with a particular emphasis on questions of responsibility. However, nowadays, higher education institutions are also seen as part of the responsibility puzzle (e.g. Cheng et al., 2022). This raises new questions, such as the extent to which the government implements ALMPs, the role of other stakeholders, such as higher education institutions, and their responses to the changing labour market values of mobility and flexibility in recent decades (‘flexicurity’). In this context, it is essential to consider not only the perspective of a single country but also cross-border perspectives, as labour markets have become increasingly global. These objectives should be considered for future research, given the changing dynamics of labour markets and policy implementation.

## Data availability statement

The datasets presented in this study can be found in online repositories: https://issp.org/; https://manifesto-project.wzb.eu/; https://www.ilo.org/. The dofiles for replicating the analyses in this study can be found at https://osf.io/xfndq/.

## Author contributions

The author confirms being the sole contributor of this work and has approved it for publication.

## Conflict of interest

The author declares that the research was conducted in the absence of any commercial or financial relationships that could be construed as a potential conflict of interest.

## Publisher’s note

All claims expressed in this article are solely those of the authors and do not necessarily represent those of their affiliated organizations, or those of the publisher, the editors and the reviewers. Any product that may be evaluated in this article, or claim that may be made by its manufacturer, is not guaranteed or endorsed by the publisher.

## Footnotes

^1^Following the concept of Rueda (2005, p. 62), labour market outsiders are not only those who are unemployed, but also those who ‘hold jobs characterized by low salaries and low levels of protection, employment rights, benefits, and social security privileges’. In this paper, I use a more rigorous definition that considers only those who are unemployed as outsiders. I will revisit this in the discussion.

^2^For a historical literature review and development of the term ‘employability’, see, e.g. McQuaid and Lindsay (2005, p. 200) or Thijssen et al. (2008, p. 167).

^3^The ISSP does not provide weights to comparable analyses across countries (for more detail, see Gesis, 2017). For robustness checks, in exceptional instances I use sampling weights provided for the level 1 variables (*cf.* Results).

^4^ISCO-08 uses a four-level hierarchical structure of occupations (a total of 436 unit groups). The highest level of aggregation is provided by the 10 major groups that were used for this analysis. For more detail, see International Labour Organization (2012).

^5^Although it might be more appropriate to measure government participation by its political composition, I nevertheless use the share of votes for social democratic parties as a proxy. Though the parties’ share of the vote does not correspond to government compositions *per se*, the influence and implications of oppositional parties is arguable visible but varies in magnitude (*cf.* Garritzmann, 2017), reflecting the population’s political sentiment.

^6^I initially added the unemployment rate. To test for multicollinearity of the participation rate, the labour productivity rate, and the unemployment rate at the macro level, I correlated the variables using Cronbach’s alpha. Based on the results, I excluded the unemployment rate, caused by high correlation with both labour force participation (α = .69) and the labour productivity rate (α = .44). For robustness checks, I added the unemployment rate while omitting the labour force participation rate, but the results hardly changed (see Supplementary Table G).

^7^Economically active population includes employed as well as unemployed persons. For instance, unemployed persons are those without work during the reference period but currently available for work, as well as job-seekers.

^8^The labour productivity rate is calculated by dividing the GDP by the number of employees.

^9^Because centring methods should be applied (*cf.* Hox et al., 2010), I use standardisation centring to use non-dimensional coefficients and make interpretation comparable.

^10^I do not add a random slope model with cross-level interaction because the focus of the research question is not how level 2 variables moderate the relationship between human capital investments and employability. Rather, I am interested in how employability varies by countries, and to explain that variability using predictors concerning ALMPs.

^11^Because the outcome variable contains factor scores with a mean of 0 and ± one standard deviation, a value of 0 means that the average score of individuals is equal to the cross-country average of employability. A value of −0.06 means about less than 0.1 standard deviation below the mean, see, e.g. Di Stefano et al. (2009).
