# Supplementary material for: Cross-country comparison: does social democratic party power increase an employee’s perceived employability?
Source: Front Sociol. 2023 Oct 3;8:1212553. doi: 10.3389/fsoc.2023.1212553 (PMC10579600; doi:10.3389/fsoc.2023.1212553)
Supplement: Supplementary file 1 [file Data_Sheet_1.docx]

Supplementary Material

**Cross-country comparison: Does social democratic party power increase employee’s perceived employability?**

Isabel M. Habicht*

*** Correspondence:** Corresponding Author: [habicht@uni-wuppertal.de](mailto:habicht@uni-wuppertal.de)

# Supplementary Figures and Tables

**Table A.** Countries included in and excluded from the dataset.

| Country | Date of collection | Date of imputation | Last vote |
| --- | --- | --- | --- |
| Australia (AU) | 17 Aug 15–14 Apr 16 | 2015 | Sep 13 |
| Austria (AT) | 09 Jul 15–16 Aug 16 | 2015 | Sep 08 |
| Belgium (BE) | 14 Oct 15–22 Mar 16 | 2015 | Jun 10 |
| *Chile (CL)* | *Apr 15–May 15* | *2015* | *–* |
| *China (CN)* | *01 Jul 15–31 Oct 15* | *2015* | *–* |
| Croatia (HR) | 01 Jul 16–26 Jul 16 | 2016 | Sep 16 |
| Czech Republic (CZ) | 31 Mar 15–20 May 15 | 2015 | Oct 13 |
| Denmark (DK) | 15 May 16–19 Jul 16 | 2016 | Sep 11 |
| Estonia (EE) | Sep 15–Oct 15 | 2015 | Mar 15 |
| Finland (FI) | 22 Sep 15–16 Dec 15 | 2015 | Apr 11 |
| France (FR) | Feb 15–Sep 15 | 2015 | Jun 12 |
| Georgia (GE) | 05 Jun 16–23 Jul 16 | 2016 | Oct 16 |
| Germany (DE) | 05 Apr 16–17 Sep 16 | 2016 | Sep 13 |
| Great Britain (GB) | 06 Jul 15–06 Nov 15 | 2015 | May 15 |
| Hungary (HU) | 16 Oct 15–23 Oct 15 | 2015 | Apr 14 |
| Iceland (IS) | 26 May 16–31 Aug 16 | 2016 | Apr 13 |
| *India (IN)* | *22 Feb 17–06 Apr 17* | *2017* | *–* |
| Israel (IL) | 23 Dec 15–05 Apr 16 | 2015 | Mar 15 |
| Japan (JP) | 24 Oct 15–01 Nov 15 | 2015 | Dec 14 |
| Latvia (LV) | 27 Aug 16–25 Sep 16 | 2016 | Oct 14 |
| Lithuania (LT) | 08 Oct 15–01 Dec 15 | 2015 | Oct 12 |
| Mexico (MX) | 10 Feb 17–03 Apr 17 | 2017 | Jul 12 |
| New Zealand (NZ) | 08 Jul 15–16 Nov 15 | 2015 | Nov 11 |
| Norway (NO) | 20 Oct 15–06 Jan 16 | 2015 | Sep 09 |
| *Philippines (PH)* | *16 Mar 16–20 Mar 16* | *2016* | *–* |
| Poland (PL) | 01 Mar 15–26 Jun 15 | 2015 | Oct 11 |
| Russia (RU) | 26 Mar 15–31 Mar 15 | 2015 | Dec 11 |
| Slovakia (SK) | 11 Sep 16–29 Oct 16 | 2016 | Mar 12 |
| Slovenia (SI) | 14 Nov 15–23 Feb 16 | 2015 | Dec 11 |
| South Africa (ZA) | 01 Oct 15–01 Mar 16 | 2015 | May 14 |
| Spain (ES) | 11 Apr 16–29 Jun 16 | 2016 | Jun 16 |
| *Suriname (SR)* | *24 Jun 15–02 Feb 17* | *2015* | *–* |
| Sweden (SE) | 01 Apr 16–11 May 16 | 2016 | Sep 10 |
| Switzerland (CH) | 12 Feb 15–10 Jul 15 | 2015 | Oct 11 |
| *Taiwan (TW)* | *08 Feb 15–01 Nov 15* | *2015* | *–* |
| United States (US) | 05 Apr 16–19 Nov 16 | 2016 | Nov 12 |
| *Venezuela (VE)* | *19 Jan 15–08 Feb 15* | *2015* | *–* |

Note: As a consequence of missing micro variables (CN, SR) and macro variables (CL, CN, IN, PH, SR, TW, VE), *countries* *in italics* are not included in the data set.

Source: (Gesis 2017), author's design.

**Table B.** Latent construct of employability, provided by the ISSP.

| Questions | Response  categories | Indicators | Factor  loadings (three items) | Factor  loadings  (two items) |
| --- | --- | --- | --- | --- |
| Q.26 How difficult or easy do you think it would be for you to find a job at least as good as your current one? | 1. Very easy  2. Fairly easy  3. Neither easy nor difficult  4. Fairly difficult  5. Very difficult  8. Can't choose  (9. No answer,  refused)  (0. Not applicable) | Respondents’ value on the labor market | .78 | .64 |
| Q.27 All in all, how likely is it that you will try to find a job with another firm or organization within the next 12 months? | 1. Very likely  2. Likely  3. Unlikely  4. Very unlikely  8. Can't choose  (9. No answer,  refused)  (0. Not applicable) | Expected exit | .79 | .64 |
| Q.28 To what extent, if at all, do you worry about the possibility of losing your job? | 1. I worry a great deal  2. I worry to some extent  3. I worry a little  4. I don't worry at all  8. Can't choose  (9. No answer,  refused)  (0. Not applicable) | Job insecurity | .02 | – |
|  |  |  | χ² = 3202.23  df = 3  KMO = .40 | χ² = 1421.921  df = 1  KMO = .50 |

Source: Jutz, Scholz and Braun (2017).

**Table C1.** Summary statistics: individual-level variables.

|  | Mean/  Prop | SD | MIN | MAX |
| --- | --- | --- | --- | --- |
| **Employability** (factor score) | 0.01 | 0.99 | -1.48 | 2.68 |
| Education |  |  |  |  |
| Upper level tertiary | 0.16 |  | 0.00 | 1.00 |
| Lower level tertiary | 0.22 |  | 0.00 | 1.00 |
| Post secondary, non-tertiary | 0.17 |  | 0.00 | 1.00 |
| Upper secondary | 0.24 |  | 0.00 | 1.00 |
| Lower secondary | 0.18 |  | 0.00 | 1.00 |
| No formal education, Primary school | 0.03 |  | 0.00 | 1.00 |
| Job Experience |  |  |  |  |
| Almost none | 0.11 |  | 0.00 | 1.00 |
| A little | 0.22 |  | 0.00 | 1.00 |
| A lot | 0.34 |  | 0.00 | 1.00 |
| Almost all | 0.32 |  | 0.00 | 1.00 |
| Further Vocational Training |  |  |  |  |
| Yes | 0.48 |  | 0.00 | 1.00 |
| No | 0.52 |  | 0.00 | 1.00 |
| Age |  |  |  |  |
| 15-25 years old | 0.09 |  | 0.00 | 1.00 |
| 26-35 years old | 0.22 |  | 0.00 | 1.00 |
| 36-45 years old | 0.25 |  | 0.00 | 1.00 |
| 46-55 years old | 0.27 |  | 0.00 | 1.00 |
| 56-65 years old | 0.17 |  | 0.00 | 1.00 |
| Gender |  |  |  |  |
| Male | 0.49 |  | 0.00 | 1.00 |
| Female | 0.51 |  | 0.00 | 1.00 |
| Relationship Status |  |  |  |  |
| Single | 0.29 |  | 0.00 | 1.00 |
| Partnership (incl. married) | 0.57 |  | 0.00 | 1.00 |
| Separated (incl. divorced and widowed) | 0.15 |  | 0.00 | 1.00 |
| Place of Living |  |  |  |  |
| Urban area | 0.30 |  | 0.00 | 1.00 |
| Small town | 0.40 |  | 0.00 | 1.00 |
| Rural area | 0.30 |  | 0.00 | 1.00 |
| Occupation |  |  |  |  |
| Services and Sales Workers | 0.17 |  | 0.00 | 1.00 |
| Managers | 0.09 |  | 0.00 | 1.00 |
| Professionals | 0.23 |  | 0.00 | 1.00 |
| Technicians | 0.16 |  | 0.00 | 1.00 |
| Clerical Support Workers | 0.08 |  | 0.00 | 1.00 |
| Agricultural Workers | 0.02 |  | 0.00 | 1.00 |
| Craft and Trades Workers | 0.11 |  | 0.00 | 1.00 |
| Plant and Machine Operators | 0.06 |  | 0.00 | 1.00 |
| Elementary Occupations | 0.08 |  | 0.00 | 1.00 |
| Employment Relationship |  |  |  |  |
| Employee | 0.89 |  | 0.00 | 1.00 |
| Self-employed | 0.11 |  | 0.00 | 1.00 |
| Meaning of Work |  |  |  |  |
| (Strongly) disagree | 0.52 |  | 0.00 | 1.00 |
| Neither agree nor disagree | 0.16 |  | 0.00 | 1.00 |
| (Strongly) agree | 0.32 |  | 0.00 | 1.00 |
| Work Situation |  |  |  |  |
| Part-time job and less (<30h) | 0.30 |  | 0.00 | 1.00 |
| Full-time job (>=30h) | 0.70 |  | 0.00 | 1.00 |
| N | 16,438 |  |  |  |

**Table C2.** Summary statistics across countries.

|  | Mean/  Prop | SD | MIN | MAX |
| --- | --- | --- | --- | --- |
| Left party power (%) | 30.72 |  | 0.00 | 68.50 |
| Left party power ^c^ | 0.00 |  | -2.09 | 2.58 |
| Labour participation rate (%) | 60.91 |  | 52.16 | 73.90 |
| Labour participation rate^c^ | 0.01 |  | -1.72 | 2.57 |
| Labour produc. rate (US-$) | 73097.54 |  | 17707.26 | 125862.24 |
| Labour productivity rate^c^ | 0.15 |  | -1.90 | 2.11 |
| N | 30 |  |  |  |

Note: Only for countries included in the analysis.

^c^ Standardized.

Source: Manifesto Project Dataset (Version 2017a), International Labour Organization (ILO).

**Table C3.** Summary Statistics by country: Share of social democratic parties, labour participation rate, and labour productivity rate.

| Country | Social democratic party (%) | Labour participation rate (%) | Labour productivity rate (US-$) |
| --- | --- | --- | --- |
| Australia | 33.383 | 64.702 | 87808.16 |
| Austria | 30.02 | 60.238 | 90788.23 |
| Belgium | 22.94 | 53.599 | 101000 |
| Croatia | 38.834 | 52.158 | 54122.33 |
| Czech Republic | 35.369 | 59.44 | 62407.46 |
| Denmark | 40.735 | 62.004 | 89010.35 |
| Estonia | 15.186 | 61.918 | 55225.8 |
| Finland | 27.235 | 58.499 | 86434.95 |
| France | 37.916 | 55.158 | 93418.69 |
| Georgia | 0 | 67.459 | 17707.26 |
| Germany | 34.3 | 60.267 | 89805.37 |
| Great Britain | 31.313 | 62.683 | 79720.41 |
| Hungary | 24.679 | 53.972 | 56894.27 |
| Iceland | 12.854 | 73.903 | 78277.61 |
| Israel | 22.604 | 64 | 75134.23 |
| Japan | 13.833 | 59.272 | 72119.47 |
| Latvia | 23.153 | 60.463 | 51111.76 |
| Lithuania | 19.176 | 59.126 | 58114.25 |
| Mexico | 66.287 | 62.279 | 38354.35 |
| New Zealand | 27.484 | 67.576 | 68034 |
| Norway | 41.579 | 64.868 | 126000 |
| Poland | 8.242 | 56.854 | 54672.22 |
| Russia | 32.953 | 63.481 | 45760.18 |
| Slovak Republic | 44.417 | 59.431 | 62372.61 |
| Slovenia | 42.3 | 57.486 | 64425.42 |
| South Africa | 68.503 | 53.03 | 44482.37 |
| Spain | 22.632 | 58.192 | 85273.8 |
| Sweden | 36.26 | 64.45 | 94533.32 |
| Switzerland | 18.722 | 68.669 | 98429.62 |
| United States | 48.736 | 61.983 | 112000 |

**Table D1.** Correlation matrix of all individual-level variables included in the study.

|  | (1) | (2) | (3) | (4) | (5) | (6) | (7) | (8) | (9) | (10) | (11) |
| --- | --- | --- | --- | --- | --- | --- | --- | --- | --- | --- | --- |
| (1) Employability |  |  |  |  |  |  |  |  |  |  |  |
| (2) Education | 0.048* |  |  |  |  |  |  |  |  |  |  |
| (3) Job experience | -0.003 | 0.181* |  |  |  |  |  |  |  |  |  |
| (4) Further training | 0.018* | 0.243* | 0.145* |  |  |  |  |  |  |  |  |
| (5) Age | -0.292* | -0.020* | 0.113* | -0.062* |  |  |  |  |  |  |  |
| (6) Gender | -0.024* | 0.080* | -0.026* | 0.032* | 0.002 |  |  |  |  |  |  |
| (7) Relationship | -0.154* | -0.021* | 0.044* | -0.042* | 0.460* | 0.103* |  |  |  |  |  |
| (8) Place of living | -0.100* | -0.112* | 0.004 | -0.009 | 0.069* | -0.019* | 0.041* |  |  |  |  |
| (9) Occupation | 0.007 | -0.538* | -0.230* | -0.241* | -0.014 | -0.150* | -0.019* | 0.109* |  |  |  |
| (10) Emp.relationship | -0.057* | -0.032* | 0.062* | -0.075* | 0.104* | -0.105* | 0.057* | 0.042* | -0.011 |  |  |
| (11) Meaning of work | -0.020* | 0.190* | 0.122* | 0.131* | 0.031* | 0.065* | -0.000 | -0.019* | -0.199* | 0.029* |  |
| (12) Work situation | -0.008 | -0.088* | -0.005 | -0.007 | -0.064* | -0.225* | -0.034* | 0.005 | 0.101* | -0.020* | -0.033* |

Significant at the .05 level, N = 16,438.

Source: ISSP 2015.

**Table D2.** Correlation matrix of all country-level variables included in the study.

|  |  |  |  |
| --- | --- | --- | --- |
|  | (1) | (2) | (3) |
| (1) Employability |  |  |  |
| (2) Left-wing party political power^a^ | 0.107* |  |  |
| (3) Labour participation rate^a^ | 0.065* | -0.236* |  |
| (4) Labour productivity rate^a^ | 0.004 | 0.043* | 0.171* |

Significant at the .05 level, M = 30.

^a^ = Standardized.

Source: ISSP 2015, Manifesto Project Dataset (Version 2017a), International Labour Organization (ILO).

**Table E.** Respondents by country and gender.

| Country | Gender | | | |
| --- | --- | --- | --- | --- |
|  | Male | Female | Total | |
| AU-Australia | 220 | 238 | 458 | |
|  | 2.75 | 2.82 | 2.79 | |
| AT-Austria | 283 | 275 | 558 | |
|  | 3.53 | 3.26 | 3.39 | |
| BE-Belgium | 426 | 419 | 845 | |
|  | 5.32 | 4.97 | 5.14 | |
| HR-Croatia | 107 | 84 | 191 | |
|  | 1.34 | 1.00 | 1.16 | |
| CZ-Czech Republic | 316 | 329 | 645 | |
|  | 3.94 | 3.91 | 3.92 | |
| DK-Denmark | 282 | 284 | 566 | |
|  | 3.52 | 3.37 | 3.44 | |
| EE-Estonia | 240 | 334 | 574 | |
|  | 3.00 | 3.96 | 3.49 | |
| FI-Finland | 219 | 242 | 461 | |
|  | 2.73 | 2.87 | 2.80 | |
| FR-France | 220 | 286 | 506 | |
|  | 2.75 | 3.39 | 3.08 | |
| GE-Georgia | 78 | 168 | 246 | |
|  | 0.97 | 1.99 | 1.50 | |
| DE-Germany | 398 | 375 | 773 | |
|  | 4.97 | 4.45 | 4.70 | |
| HU-Hungary | 182 | 250 | 432 | |
|  | 2.27 | 2.97 | 2.63 | |
| IS-Iceland | 227 | 289 | 516 | |
|  | 2.83 | 3.43 | 3.14 | |
| IL-Israel | 300 | 302 | 602 | |
|  | 3.74 | 3.58 | 3.66 | |
| JP-Japan | 341 | 292 | 633 | |
|  | 4.26 | 3.47 | 3.85 | |
| LV-Latvia | 232 | 259 | 491 | |
|  | 2.90 | 3.07 | 2.99 | |
| LT-Lithuania | 160 | 181 | 341 | |
|  | 2.00 | 2.15 | 2.07 | |
| MX-Mexico | 292 | 176 | 468 | |
|  | 3.64 | 2.09 | 2.85 | |
| NZ-New Zealand | 142 | 154 | 296 | |
|  | 1.77 | 1.83 | 1.80 | |
| NO-Norway | 330 | 371 | 701 | |
|  | 4.12 | 4.40 | 4.26 | |
| PL-Poland | 282 | 286 | 568 | |
|  | 3.52 | 3.39 | 3.46 | |
| RU-Russia | 286 | 255 | 541 | |
|  | 3.57 | 3.03 | 3.29 | |
| SK-Slovak Republic | 193 | 260 | 453 | |
|  | 2.41 | 3.09 | 2.76 | |
| SI-Slovenia | 205 | 198 | 403 | |
|  | 2.56 | 2.35 | 2.45 | |
| ZA-South Africa | 319 | 312 | 631 | |
|  | 3.98 | 3.70 | 3.84 | |
| ES-Spain | 354 | 347 | 701 | |
|  | 4.42 | 4.12 | 4.26 | |
| SE-Sweden | 245 | 286 | 531 | |
|  | 3.06 | 3.39 | 3.23 | |
| CH-Switzerland | 355 | 359 | 714 | |
|  | 4.43 | 4.26 | 4.34 | |
| GB-Great Britain | 361 | 404 | 765 | |
|  | 4.51 | 4.80 | 4.65 | |
| US-United States | 418 | 410 | 828 | |
|  | 5.22 | 4.87 | 5.04 | |
| Total | 8013 | 8425 | 16438 | |
|  | 100.00 | 100.00 | 100.00 | |
| Source: ISSP 2015. | | | |  |

**Table F.** Results of the Multilevel Analysis including EPL.

|  | (1) | (2) | (3) | (4) | (5) | (6) | (7) |
| --- | --- | --- | --- | --- | --- | --- | --- |
|  | Variance -  component model | Random intercept models | | | | Only women | Only men |
| Constant | -0.02 | 0.69^***^ | 0.73^***^ | 0.71^***^ | 0.72^***^ | 0.71^***^ | 0.71^***^ |
|  | (0.05) | (0.06) | (0.07) | (0.07) | (0.06) | (0.07) | (0.09) |
| **FIXED: LEVEL 1 Education** |  |  |  |  |  |  |  |
| Upper level tertiary *(Reference)* |  |  |  |  |  |  |  |
| Lower level tertiary |  |  | -0.08^**^ | -0.08^**^ | -0.08^**^ | -0.08^*^ | -0.07 |
|  |  |  | (0.03) | (0.03) | (0.03) | (0.04) | (0.04) |
| Post secondary, non-tertiary |  |  | -0.14^***^ | -0.14^***^ | -0.15^***^ | -0.20^***^ | -0.09 |
|  |  |  | (0.03) | (0.03) | (0.03) | (0.04) | (0.05) |
| Upper secondary |  |  | -0.16^***^ | -0.16^***^ | -0.17^***^ | -0.20^***^ | -0.12^**^ |
|  |  |  | (0.03) | (0.03) | (0.03) | (0.04) | (0.05) |
| Lower secondary |  |  | -0.18^***^ | -0.18^***^ | -0.18^***^ | -0.25^***^ | -0.12^*^ |
|  |  |  | (0.03) | (0.03) | (0.03) | (0.05) | (0.05) |
| No formal education^a^ |  |  | -0.27^***^ | -0.28^***^ | -0.28^***^ | -0.24^**^ | -0.29^***^ |
|  |  |  | (0.06) | (0.06) | (0.06) | (0.09) | (0.08) |
| **Job Experience** |  |  |  |  |  |  |  |
| Almost none *(Reference)* |  |  |  |  |  |  |  |
| A little |  |  | 0.14^***^ | 0.14^***^ | 0.14^***^ | 0.17^***^ | 0.09^*^ |
|  |  |  | (0.03) | (0.03) | (0.03) | (0.04) | (0.05) |
| A lot |  |  | 0.10^***^ | 0.10^***^ | 0.10^***^ | 0.12^**^ | 0.07 |
|  |  |  | (0.03) | (0.03) | (0.03) | (0.04) | (0.04) |
| Almost all |  |  | 0.12^***^ | 0.12^***^ | 0.12^***^ | 0.13^**^ | 0.08 |
|  |  |  | (0.03) | (0.03) | (0.03) | (0.04) | (0.04) |
| **Further vocational training** |  |  | -0.01 | -0.01 | -0.01 | 0.01 | -0.03 |
|  |  |  | (0.02) | (0.02) | (0.02) | (0.02) | (0.02) |
| **FIXED: LEVEL 2** |  |  |  |  |  |  |  |
| Left party power(std) |  |  |  | 0.10^**^ | 0.13^***^ | 0.14^***^ | 0.12^**^ |
|  |  |  |  | (0.04) | (0.03) | (0.03) | (0.04) |
| Participation(std) |  |  |  |  | 0.12^**^ | 0.12^***^ | 0.12^**^ |
|  |  |  |  |  | (0.04) | (0.04) | (0.04) |
| Labour productivity(std) |  |  |  |  | -0.02 | -0.00 | -0.04 |
|  |  |  |  |  | (0.04) | (0.03) | (0.04) |
| EPLex(std) |  |  |  |  | -0.06 | -0.08^**^ | -0.04 |
|  |  |  |  |  | (0.03) | (0.03) | (0.04) |
| *AIC* | 38043.37 | 36933.87 | 36937.45 | 36937.15 | 36946.39 | 18823.49 | 18284.13 |
| *BIC* | 38065.95 | 37114.53 | 37185.85 | 37193.07 | 37224.89 | 19070.35 | 18529.31 |
| M (country) |  | 24 | 24 | 24 | 24 | 24 | 24 |
| R² (individual)^b^ |  | 0.092 | 0.096 | 0.106 | 0.117 | 0.138 | 0.101 |
| R² (country)^b^ |  | 0.188 | 0.184 | 0.378 | 0.576 | 0.680 | 0.457 |
| df | 0 | 21 | 30 | 31 | 34 | 33 | 33 |
| N (individual) | 13729 | 13729 | 13729 | 13729 | 13729 | 7024 | 6705 |

Note: Estimations from the random intercept model with individual- and group-level effects (REML). Standard errors in parentheses.

^*^ *p* < 0.05, ^**^ *p* < 0.01, ^***^ *p* < 0.001. ^a^ Includes primary school. ^b^ Variance explained by proportional reduction in prediction error (Snijders and Bosker 1994, 350–54; Snijders and Bosker 1999, 99–105).

**Table G.** Results of the Multilevel Analysis including unemployment rate.

|  | (1) | (2) | (3) | (4) | (5) | (6) | (7) |
| --- | --- | --- | --- | --- | --- | --- | --- |
|  | Variance -  component model | Random intercept models | | | | Only women | Only men |
| Constant | -0.02 | 0.69^***^ | 0.73^***^ | 0.71^***^ | 0.72^***^ | 0.71^***^ | 0.71^***^ |
|  | (0.05) | (0.06) | (0.07) | (0.07) | (0.07) | (0.08) | (0.09) |
| **FIXED: LEVEL 1 Education** |  |  |  |  |  |  |  |
| Upper level tertiary *(Reference)* |  |  |  |  |  |  |  |
| Lower level tertiary |  |  | -0.08^**^ | -0.08^**^ | -0.08^**^ | -0.08^*^ | -0.07 |
|  |  |  | (0.03) | (0.03) | (0.03) | (0.04) | (0.04) |
| Post secondary, non-tertiary |  |  | -0.14^***^ | -0.14^***^ | -0.14^***^ | -0.19^***^ | -0.08 |
|  |  |  | (0.03) | (0.03) | (0.03) | (0.04) | (0.05) |
| Upper secondary |  |  | -0.16^***^ | -0.16^***^ | -0.16^***^ | -0.20^***^ | -0.12^*^ |
|  |  |  | (0.03) | (0.03) | (0.03) | (0.04) | (0.05) |
| Lower secondary |  |  | -0.18^***^ | -0.18^***^ | -0.18^***^ | -0.25^***^ | -0.13^*^ |
|  |  |  | (0.03) | (0.03) | (0.03) | (0.05) | (0.05) |
| No formal education^a^ |  |  | -0.27^***^ | -0.28^***^ | -0.28^***^ | -0.23^**^ | -0.28^***^ |
|  |  |  | (0.06) | (0.06) | (0.06) | (0.09) | (0.08) |
| **Job Experience** |  |  |  |  |  |  |  |
| Almost none *(Reference)* |  |  |  |  |  |  |  |
| A little |  |  | 0.14^***^ | 0.14^***^ | 0.14^***^ | 0.17^***^ | 0.09^*^ |
|  |  |  | (0.03) | (0.03) | (0.03) | (0.04) | (0.05) |
| A lot |  |  | 0.10^***^ | 0.10^***^ | 0.10^***^ | 0.12^**^ | 0.07 |
|  |  |  | (0.03) | (0.03) | (0.03) | (0.04) | (0.04) |
| Almost all |  |  | 0.12^***^ | 0.12^***^ | 0.12^***^ | 0.13^**^ | 0.08 |
|  |  |  | (0.03) | (0.03) | (0.03) | (0.04) | (0.04) |
| **Further vocational training** |  |  | -0.01 | -0.01 | -0.01 | 0.01 | -0.03 |
|  |  |  | (0.02) | (0.02) | (0.02) | (0.02) | (0.02) |
| **FIXED: LEVEL 2** |  |  |  |  |  |  |  |
| Left party power(std) |  |  |  | 0.10^**^ | 0.11^**^ | 0.12^**^ | 0.10^*^ |
|  |  |  |  | (0.04) | (0.04) | (0.04) | (0.04) |
| Labour productivity(std) |  |  |  |  | -0.00 | 0.02 | -0.03 |
|  |  |  |  |  | (0.04) | (0.05) | (0.05) |
| Unemployment rate(std) |  |  |  |  | -0.03 | -0.03 | -0.03 |
|  |  |  |  |  | (0.04) | (0.04) | (0.04) |
| *AIC* | 38043.37 | 36933.87 | 36937.45 | 36937.15 | 36949.70 | 18830.41 | 18284.67 |
| *BIC* | 38065.95 | 37114.53 | 37185.85 | 37193.07 | 37220.68 | 19070.41 | 18523.05 |
| M (country) |  | 24 | 24 | 24 | 24 | 24 | 24 |
| R² (individual)^b^ |  | 0.092 | 0.096 | 0.106 | 0.104 | 0.120 | 0.091 |
| R² (country)^b^ |  | 0.188 | 0.184 | 0.378 | 0.340 | 0.398 | 0.280 |
| df | 0 | 21 | 30 | 31 | 33 | 32 | 32 |
| N (individual) | 13729 | 13729 | 13729 | 13729 | 13729 | 7024 | 6705 |

Note: Estimations from the random intercept model with individual- and group-level effects (REML). Standard errors in parentheses.

* p < 0.05, ** p < 0.01, *** p < 0.001. a Includes primary school. b Variance explained by proportional reduction in prediction error (Snijders and Bosker 1994:350–54; Snijders and Bosker 1999:99–105).

**Table H.** Multilevel results of employee and country-level variables on perceived employability

|  | (1) | (2) | (3) | (4) | (5) | (6) | (7) |
| --- | --- | --- | --- | --- | --- | --- | --- |
|  | Variance - component model | Random intercept models | | | | | |
|  |  |  |  |  |  | Only women | Only men |
| Constant | 0.01 | 0.71^***^ | 0.72^***^ | 0.72^***^ | 0.72^***^ | 0.73^***^ | 0.69^***^ |
|  | (0.04) | (0.05) | (0.06) | (0.06) | (0.06) | (0.07) | (0.08) |
| **FIXED: LEVEL 1 Education** |  |  |  |  |  |  |  |
| Upper level tertiary |  |  | *(Reference)* | | | | |
| Lower level tertiary |  |  | -0.06^*^ | -0.06^*^ | -0.06^*^ | -0.05 | -0.07 |
|  |  |  | (0.02) | (0.02) | (0.02) | (0.03) | (0.04) |
| Post secondary, non-tertiary |  |  | -0.12^***^ | -0.12^***^ | -0.12^***^ | -0.16^***^ | -0.07 |
|  |  |  | (0.03) | (0.03) | (0.03) | (0.04) | (0.04) |
| Upper secondary |  |  | -0.15^***^ | -0.15^***^ | -0.15^***^ | -0.16^***^ | -0.13^**^ |
|  |  |  | (0.03) | (0.03) | (0.03) | (0.04) | (0.04) |
| Lower secondary |  |  | -0.17^***^ | -0.18^***^ | -0.18^***^ | -0.23^***^ | -0.13^**^ |
|  |  |  | (0.03) | (0.03) | (0.03) | (0.04) | (0.05) |
| No formal education^a^ |  |  | -0.25^***^ | -0.26^***^ | -0.26^***^ | -0.16^*^ | -0.31^***^ |
|  |  |  | (0.05) | (0.05) | (0.05) | (0.08) | (0.07) |
| **Job Experience** |  |  |  |  |  |  |  |
| Almost none |  |  | *(Reference)* | | | | |
| A little |  |  | 0.15^***^ | 0.15^***^ | 0.15^***^ | 0.20^***^ | 0.09^*^ |
|  |  |  | (0.03) | (0.03) | (0.03) | (0.04) | (0.04) |
| A lot |  |  | 0.13^***^ | 0.13^***^ | 0.13^***^ | 0.15^***^ | 0.09^*^ |
|  |  |  | (0.03) | (0.03) | (0.03) | (0.03) | (0.04) |
| Almost all |  |  | 0.14^***^ | 0.14^***^ | 0.14^***^ | 0.17^***^ | 0.08^*^ |
|  |  |  | (0.03) | (0.03) | (0.03) | (0.04) | (0.04) |
| **Further vocational training** |  |  | -0.01 | -0.01 | -0.01 | -0.01 | -0.03 |
|  |  |  | (0.02) | (0.02) | (0.02) | (0.02) | (0.02) |
| **Age** |  |  |  |  |  |  |  |
| 15-25 |  | *(Reference)* | | | | | |
| 26-35 |  | -0.22^***^ | -0.24^***^ | -0.24^***^ | -0.24^***^ | -0.25^***^ | -0.22^***^ |
|  |  | (0.03) | (0.03) | (0.03) | (0.03) | (0.04) | (0.04) |
| 36-45 |  | -0.38^***^ | -0.40^***^ | -0.40^***^ | -0.40^***^ | -0.41^***^ | -0.38^***^ |
|  |  | (0.03) | (0.03) | (0.03) | (0.03) | (0.04) | (0.05) |
| 46-55 |  | -0.58^***^ | -0.60^***^ | -0.60^***^ | -0.60^***^ | -0.60^***^ | -0.59^***^ |
|  |  | (0.03) | (0.03) | (0.03) | (0.03) | (0.04) | (0.05) |
| 56-65 |  | -0.84^***^ | -0.85^***^ | -0.85^***^ | -0.85^***^ | -0.87^***^ | -0.83^***^ |
|  |  | (0.03) | (0.03) | (0.03) | (0.03) | (0.05) | (0.05) |
| **Female** |  | -0.06^***^ | -0.05^***^ | -0.05^***^ | -0.05^***^ |  |  |
|  |  | (0.02) | (0.02) | (0.02) | (0.02) |  |  |
| **Relationship status** |  |  |  |  |  |  |  |
| Single |  | *(Reference)* | | | | | |
|  |  |  |  |  |  |  |  |
| Married, Partnership |  | -0.12^***^ | -0.12^***^ | -0.12^***^ | -0.12^***^ | -0.15^***^ | -0.09^**^ |
|  |  | (0.02) | (0.02) | (0.02) | (0.02) | (0.03) | (0.03) |
| Separated, Divorced, Widowed |  | -0.08^**^ | -0.07^**^ | -0.07^**^ | -0.07^**^ | -0.12^***^ | -0.01 |
|  |  | (0.03) | (0.03) | (0.03) | (0.03) | (0.03) | (0.04) |
| **Place of living** |  |  |  |  |  |  |  |
| Urban area |  | *(Reference)* | | | | | |
|  |  |  |  |  |  |  |  |
| Small town |  | -0.10^***^ | -0.09^***^ | -0.09^***^ | -0.09^***^ | -0.13^***^ | -0.05 |
|  |  | (0.02) | (0.02) | (0.02) | (0.02) | (0.03) | (0.03) |
| Rural area |  | -0.13^***^ | -0.11^***^ | -0.11^***^ | -0.11^***^ | -0.16^***^ | -0.06 |
|  |  | (0.02) | (0.02) | (0.02) | (0.02) | (0.03) | (0.03) |
| **Occupation** |  |  |  |  |  |  |  |
| Services and Sales Workers |  | *(Reference)* | | | | | |
|  |  |  |  |  |  |  |  |
| Managers |  | -0.07^*^ | -0.13^***^ | -0.13^***^ | -0.13^***^ | -0.13^**^ | -0.12^*^ |
|  |  | (0.03) | (0.03) | (0.03) | (0.03) | (0.05) | (0.05) |
| Professionals |  | -0.04 | -0.12^***^ | -0.12^***^ | -0.12^***^ | -0.17^***^ | -0.06 |
|  |  | (0.02) | (0.03) | (0.03) | (0.03) | (0.03) | (0.04) |
| Technicians and Associate Professionals |  | -0.08^**^ | -0.12^***^ | -0.12^***^ | -0.12^***^ | -0.13^***^ | -0.11^*^ |
|  |  | (0.03) | (0.03) | (0.03) | (0.03) | (0.03) | (0.04) |
| Clerical Support Workers |  | -0.13^***^ | -0.15^***^ | -0.15^***^ | -0.15^***^ | -0.20^***^ | -0.07 |
|  |  | (0.03) | (0.03) | (0.03) | (0.03) | (0.04) | (0.06) |
| Skilled Agricultural, Forestry and Fishery Workers |  | -0.10 | -0.09 | -0.09 | -0.09 | 0.13 | -0.17^*^ |
|  |  | (0.06) | (0.06) | (0.06) | (0.06) | (0.10) | (0.07) |
| Craft and Related Trades Workers |  | -0.01 | -0.01 | -0.01 | -0.01 | -0.07 | -0.01 |
|  |  | (0.03) | (0.03) | (0.03) | (0.03) | (0.06) | (0.04) |
| Plant and Machine Operators and Assemblers |  | -0.05 | -0.04 | -0.03 | -0.03 | -0.17^*^ | -0.01 |
|  |  | (0.04) | (0.04) | (0.04) | (0.04) | (0.08) | (0.05) |
| Elementary Occupations |  | -0.03 | -0.00 | -0.00 | -0.00 | 0.03 | -0.04 |
|  |  | (0.03) | (0.03) | (0.03) | (0.03) | (0.04) | (0.05) |
| **Self-employed** |  | -0.10^***^ | -0.10^***^ | -0.10^***^ | -0.10^***^ | -0.15^***^ | -0.07^*^ |
|  |  | (0.02) | (0.02) | (0.02) | (0.02) | (0.04) | (0.03) |
| **Meaning of work** |  |  |  |  |  |  |  |
| (Strongly) Disagree |  | *(Reference)* | | | | | |
|  |  |  |  |  |  |  |  |
| Neither agree nor disagree |  | 0.05^*^ | 0.06^**^ | 0.06^**^ | 0.06^**^ | 0.05 | 0.08^*^ |
|  |  | (0.02) | (0.02) | (0.02) | (0.02) | (0.03) | (0.03) |
| (Strongly) agree |  | 0.04^*^ | 0.06^***^ | 0.06^***^ | 0.06^***^ | 0.05^*^ | 0.07^**^ |
|  |  | (0.02) | (0.02) | (0.02) | (0.02) | (0.03) | (0.03) |
| **Full-time job** (>30h) |  | -0.05^**^ | -0.04^**^ | -0.04^**^ | -0.04^*^ | -0.03 | -0.07^**^ |
|  |  | (0.02) | (0.02) | (0.02) | (0.02) | (0.02) | (0.03) |
| **FIXED: LEVEL 2** |  |  |  |  |  |  |  |
| Left party power(std) |  |  |  | 0.07^*^ | 0.10^**^ | 0.11^***^ | 0.09^**^ |
|  |  |  |  | (0.03) | (0.03) | (0.03) | (0.03) |
| Participation(std) |  |  |  |  | 0.11^**^ | 0.12^***^ | 0.11^**^ |
|  |  |  |  |  | (0.03) | (0.03) | (0.04) |
| Labour productivity(std) |  |  |  |  | -0.01 | 0.02 | -0.05 |
|  |  |  |  |  | (0.04) | (0.04) | (0.04) |
| **RANDOM PART** |  |  |  |  |  |  |  |
| Variance components: |  |  |  |  |  |  |  |
| Individual | .93 | .85 | .85 | .85 | .85 | .83 | .86 |
|  | (.01) | (.01) | (.01) | (.01) | (.01) | (.01) | (.01) |
| Country | .05 | .04 | .04 | .03 | .03 | .03 | .03 |
|  | (.01) | (.01) | (.01) | (.01) | (.01) | (.01) | (.01) |
| *AIC* | 45589.34 | 44225.65 | 44214.30 | 44217.35 | 44221.45 | 22600.32 | 21787.88 |
| *BIC* | 45612.47 | 44410.63 | 44468.64 | 44479.40 | 44498.91 | 22846.69 | 22032.49 |
| M (country) |  | 30 | 30 | 30 | 30 | 30 | 30 |
| R² (individual)^b^ |  | 0.092 | 0.095 | 0.099 | 0.108 | 0.124 | 0.097 |
| R² (country)^b^ |  | 0.173 | 0.172 | 0.252 | 0.430 | 0.491 | 0.372 |
| df | 0 | 21 | 30 | 31 | 33 | 32 | 32 |
| N (individual) | 16438 | 16438 | 16438 | 16438 | 16438 | 8425 | 8013 |

Note: Estimations from the random intercept model with individual- and group-level effects (REML). Standard errors in parentheses.

^*^ *p* < 0.05, ^**^ *p* < 0.01, ^***^ *p* < 0.001.

^a^ Includes primary school.

^b^ Variance explained by proportional reduction in prediction error (Snijders and Bosker 1994, 350–54; Snijders and Bosker 1999, 99–105).
